# Attitudes of medical students in Croatia toward rural medicine education and practice

**DOI:** 10.3389/fmed.2025.1485790

**Published:** 2025-02-07

**Authors:** Nataša Mrduljaš-Ðujić, Ivana Radić, Nina Bašić Marković, Toni Vrgoč, Maja Buljubašić

**Affiliations:** ^1^Specialist Practice in Family Medicine Postira Nataša Mrduljaš-Ðujić, Postira, Island of Brač, Croatia; ^2^Department of Family Medicine, Faculty of Medicine, University of Split, Split, Croatia; ^3^Ethos, Consultancy and Education Business, Algebra University College, Zagreb, Croatia; ^4^Institution for Primary Health Care Srdoči, Rijeka, Croatia; ^5^Faculty of Medicine, University of Rijeka, Rijeka, Croatia; ^6^Ordinacija Obiteljske Medicine Maja Buljubašić, Zagreb, Croatia

**Keywords:** rural medicine, family doctor, family medicine, rural curriculum, medical students

## Abstract

**Introduction:**

Recruiting and retaining doctors in rural areas is challenging. In Croatia, medical school curricula lack content on rural medicine and specialized training for rural practice. This study explores the opinions and attitudes of first- and sixth-year medical students in all four medical schools in Croatia regarding working in rural areas.

**Methods:**

An online questionnaire was administered to Croatian medical students in their first and final years between January 2022 and February 2023. Responses were obtained from 690 participants from the Universities of Osijek, Rijeka, Split, and Zagreb. The cross-sectional study included 13 questions, with 5 on socio-demographic data. Data were analyzed using descriptive statistics and non-parametric tests (chi-square) to assess group differences.

**Results:**

Compared to first-year students, final-year students feel less prepared by their education for rural practice (χ^2^ = 84.287; *P* = 0.000) but are more interested in working in rural areas (χ^2^ = 26.810; *P* = 0.000). Most students believe rural doctors need additional financial incentives, with this belief significantly stronger among final-year students (χ^2^ = 14.192; *P* = 0.000). Both groups agree that rural doctors face poor working conditions (χ^2^ = 1.524; *P* = 0.217). No statistically significant differences were found regarding job interest outside city centers (χ^2^ = 2.041; *P* = 0.564) or choosing rural medical practice (χ^2^ = 4.795; *P* = 0.187) among medical students from the Universities of Osijek, Rijeka, Split, and Zagreb. Students from rural settlements were more often interested in jobs outside the city center (72.1%) compared to those from smaller towns (60.6%), [χ^2^(1) = 5.142, *p* = 0.023] and larger cities (44.1%), [χ^2^(1) = 28.978, *p* = 0.000].

**Conclusion:**

Although Croatian medical students show interest in working in rural areas, their education lacks sufficient preparation for the unique challenges of rural practice. They view the current conditions for rural doctors as inadequate and believe that additional financial incentives are necessary. Interest in rural practice is consistent across medical faculties in Croatia, with students living in rural areas showing a higher interest in working there.

## 1 Introduction

Access to adequate medical care is a fundamental human right, as established by the United Nations Charter of 1948. Implementing primary health care stands as one of the most significant systemic and ideological health reforms of modern times, resulting in more efficient and equitable healthcare systems in countries where it is well-developed (1). The World Health Organization highlights the challenges in recruiting and retaining doctors in rural areas, emphasizing that the availability of health workers is crucial for the health of rural populations (2, 3).

Rural areas, characterized by diverse cultures, economic challenges, and traditions, require special consideration in medical education (4, 5). Providing healthcare education in rural settings demands a distinct educational approach, equipping medical students with specific skills and diagnostic-therapeutic procedures tailored to these communities (6). Physicians in rural settings face numerous challenges, often with limited resources and minimal social support (7, 8). Rural areas are often faced with a lack of diagnostic equipment and medical supplies, as well as access to specialists (9, 10). Distance from rural areas makes large medical centers difficult to access, especially when combined with scarce public transportation (10–12). Rural areas can also have poorer access to healthcare due to geographical attributes; areas could be inaccessible due to terrain and houses can be scattered far apart (11). The demographics of rural areas are also problematic because rural areas tend to have an older population (11, 13). Positive predictors for working in rural areas include growing up in such environments and having part of one's education conducted there. Conversely, negative predictors encompass lack of employment opportunities for partners/spouses, inadequate opportunities for children's development, perceived infrastructure deficits, heavy workloads, social isolation, and limited professional development (14–17). Personal experience and exposure to rural work environments with positive, friendly relationships with patients further motivate students. These insights can guide educators and policymakers in structuring effective educational experiences and motivating young doctors (18).

Integrating rural clinical experiences into medical curricula is essential for the education of Twenty first-century medical professionals (19). Educating medical students in rural settings, where they assume more inclusive roles compared to urban education, has demonstrated higher levels of satisfaction, knowledge, skills, attitudes, and readiness to assume the role of a physician (18, 20, 21). This decentralized education model benefits both students and rural health units. While it requires significant effort and resources from educational institutions, health institutions, and students and their mentors, it provides students with broader education, opportunities for specialization, increased self-confidence, and a more complex professional identity. It also enhances the likelihood of students continuing to work in rural settings (18, 20–23).

Croatia has four medical faculties located in its largest cities: Rijeka and Split along the coast, and Zagreb and Osijek inland. Family Medicine is a required course in the sixth year of the Integrated Medicine program at all four schools, incorporating lectures, seminars, and practical exercises. While Osijek emphasizes lectures, Zagreb focuses on hands-on exercises, and Split prioritizes seminars, none of the curricula specifically address rural medicine, despite Split offering some rural practice opportunities on islands. However, the importance of family medicine in Croatia is also evident in the curricula of medical faculties, where a large number of hours in family medicine courses are dedicated to practical training in the offices of family physicians (24–27).

Medical education in Croatia spans 6 years (28). Upon graduation, newly qualified doctors typically work as substitutes for family physicians or in emergency medicine while awaiting their specialization. During this interim period, they have the opportunity to choose between rural and urban locations for their work assignments. One possible explanation for Croatian medical students' interest in rural practice is the point-based system used when applying for specialization. This system evaluates candidates based on academic performance, scientific publications, awards, work experience, and a job interview (29). Notably, work experience in rural areas—defined as regions with a development index below 100%—is weighted twice as highly as experience in urban settings. This incentivizes rural practice by offering a clear career advantage (27, 29). In Croatia, however, the criteria for defining rural medical practice primarily focus on the population size of a settlement, its distance from the nearest hospital, and the accessibility of healthcare services. Rural areas of the Republic of Croatia, which make up more than 90% of its land area and are home to approximately 47% of the total population. Taking into account some data from the literature, rural settlements were defined with fewer than 5,000 inhabitants (30, 31).

In the Croatian healthcare system, patients can consult their family physician directly, without needing a referral, although appointments may sometimes be required (32). If necessary, family physicians, as gate keepers, provide referrals to hospital specialists. Hospitals are predominantly located in larger urban centers, whereas family physicians are distributed across both urban and rural areas (33, 34). However, more remote areas face a shortage of healthcare providers. In particular, unmet medical needs due to geographical distance in Croatia were higher than in any other EU Member State (0.7% of the population, with an EU average of 0.1 %), and unmet needs were higher among older people (35).

We hypothesized that attitudes of first-year and final-year medical students to work in rural areas would differ due to their experience training in the medical field. These experiences likely influence their interest in specific career paths, including rural medicine (36–38). This study aimed to explore whether preferences for rural medicine vary between students at these two stages of medical education.

### 1.1 Hypotheses

1. There are differences in the attitudes of first-year and final-year medical students toward working in rural areas. Final year students will have more positive attitudes toward working in rural areas.
2. There is a difference in considering the choice of rural medical practice among students from different faculties.
3. There is a difference in the choice of rural medical practice among students from different places of origin. Students from rural areas are more likely to consider working in rural areas.

The aim of this study is to explore the opinions and attitudes of first- and sixth-year medical students regarding working in rural areas, as well as to identify potential differences in these attitudes among the various faculties. The purpose is to understand medical students' intent to go rural and what could influence this to happen.

## 2 Materials and methods

A cross-sectional research design was used in this study. The research was conducted in the period from January 2022 to February 2023. A web-based survey was used to collect data and the survey link was sent to first and sixth year undergraduate medical students of the Universities of Split, Rijeka, Osijek, and Zagreb (*N* = 2,472) via e mail. Responses were obtained from 690 participants. The authors did not send reminders to potential participants.

Participants completed the questionnaire by clicking the relevant link.

The overall response rate was 27.91 %. Based on the online sample size calculation for this response rate (95% CI ± 5%) from a total population of 2,472, the minimum required sample size is 286 (39).

Online questionnaire consisted of 12 questions created through Google Forms. The questionnaire consisted of a part that includes socio-demographic data (five questions) and seven questions related to rural medicine.

Most of the participants were female, which is in line with the ratio of men to women in the population of medical students in Croatia(40). The age of the participants ranged between 17 and 31 years, with an average age of 22 years (*M* = 22.03; SD = 2.815). The intention was to collect their place of origin and settlements which are categorized based on population size: those with fewer than 5,000 inhabitants are classified as rural, those with populations between 5,000 and 20,000 are considered small towns, and those with populations exceeding 20,000 are designated as larger cities. The structure of the sample is shown in Table 1.

**Table 1:** Descriptive statistical indicators (frequencies and percentages) for demographic variables (*N* = 690).

**Variable**		***f***	**%**
Gender	Male	181	26.2
	Female	509	73.8
Settlement you come from	Rural	136	19.7
	Small town	264	38.3
	Bigger city	290	42.0
University	Osijek	57	8.3
	Rijeka	162	23.5
	Split	181	26.2
	Zagreb	290	42.0
Year of study	1^st^ (first)	323	46.8
	6^th^ (sixth)	367	53.2

This study was approved by the Ethics Committee of the University of Split, School of Medicine. Electronic informed consent was obtained from all participants prior to starting the research.

## 3 Results

The results of the research were processed by the statistical program SPSS for Windows for personal computers, version 23.0. In addition to basic descriptive statistics, appropriate non-parametric statistical procedures were also used. The chi-square test (X^2^) was used to test differences between groups. As a criterion of significance, a criterion of 5% risk of error was used. Based on the sample size formula for comparing two proportions (two-tailed) with a 95% confidence interval, the power of the sample size is 99.9% (41).

First, descriptive statistics are presented, followed by results addressing the research questions (Tables 2, 3).

**Table 2:** Number and structure of participants (students) at each faculty.

**Medical faculties**	**Years of study (total number of students)**			**Years of study (number of students participating in the research)**		
	**1** ^st^	**6** ^th^	**Total**	**1** ^st^	**6** ^th^	**Total**
Split	182	179	361	68 (37.36%)	113 (63.13%)	181 (50.14%)
Rijeka	273	266	539	87 (31.87%)	75 (28.2%)	162 (30.06%)
Zagreb	693	605	1,298	137 (19.77%)	153 (25.29%)	290 (22.34%)
Osijek	144	130	274	31 (21.52%)	26 (20%)	57 (20.8%)
Total	1,292	1,180	2,472	323 (25%)	367 (31.1%)	690 (27.91%)

**Table 3:** Descriptive statistical indicators (frequencies and percentages) for all dependent variables measured in the study (*N* = 690).

**Variable**		***f***	**%**
Would you consider family medicine among the medical professions you would choose as a career?	yes	313	45.4
	no	377	54.6
Are you interested in a job outside of (a major) urban center?	yes	386	55.9
	no	304	44.1
Do you believe that the undergraduate program provides the knowledge and skills necessary for working in a rural area?	yes	308	44.6
	no	382	55.4
Have you considered working in rural areas?	yes	386	55.9
	no	304	44.1
Do you believe that rural doctors have good working conditions?	yes	101	14.6
	no	589	85.4
Do you believe that the work of rural doctors should be financially incentivized compared to urban doctors?	yes	630	91.3
	no	60	8.7

The first problem of this research was to examine whether there is a difference in attitudes toward working in rural areas between first-year and sixth-year (final year) medical students. To address this issue, we calculated the chi-square test between the group of first-year and final-year medical students (Table 4).

**Table 4:** Descriptive statistical indicators (frequencies and percentages) and the results of the chi-square test conducted between first-year (*N* = 323) and final-year (*N* = 367) medical students for all variables related to attitudes toward working in rural areas.

**Variable**		**1^st^ year**	**6^th^ year**	***χ^2^***	***P***
Would you consider family medicine among the medical professions you would choose as a career?	yes	129	184	7.209	0.007
		(39.9%)	(50.1%)		
	no	194	183		
		(60.1%)	(49.9%)		
Are you interested in a job outside of (a major) urban center?	yes	168	218	3.805	0.051
		(52.0%)	(59.4%)		
	no	155	149		
		(48.0%)	(40.6%)		
Do you believe that the undergraduate program provides the knowledge and skills necessary for working in a rural area?	yes	204	104	84.287	0.000
		(63.2%)	(28.3%)		
	no	119	263		
		(36.8%)	(71.7%)		
Have you considered working in rural areas?	yes	147	239	26.810	0.000
		(45.5%)	(65.1%)		
	no	176	128		
		(54.5%)	(34.9%)		
Do you believe that rural doctors have good working conditions?	yes	53	48	1.524	0.217
		(16.4%)	(13.1%)		
	no	270	319		
		(83.6%)	(86.9%)		
Do you believe that the work of rural doctors should be financially incentivized compared to urban doctors?	yes	281	349	14.192	0.000
		(87.0%)	(95.1%)		
	no	42	18		
		(13.0%)	(4.9%)		

A significantly higher percentage of sixth-year medical students, compared to first-year students, view family medicine as a potential career choice (*P* = 0.007).

First-year medical students are significantly more likely than final-year students to believe that the undergraduate program equips them with the knowledge and skills needed to work in rural areas (*P* = 0.000).

Among first-year medical students, a significantly lower proportion has considered working in rural areas compared to their final-year counterparts (*P* = 0.000).

A significantly higher proportion of final-year students, unlike their first-year counterparts, believe that rural doctors should receive additional financial incentives compared to urban doctors (*P* = 0.000).

The difference in interest between first-year and final-year students in working outside of major urban centers suggests a trend where a greater number of final-year students are inclined to seek employment in these areas compared to first-year students (*P* = 0.051).

Most first-year and final-year students believe that rural doctors face poor working conditions (*P* = 0.217).

The second objective of this research was to investigate differences between faculties regarding the choice of medical practice in rural areas. To achieve this, we conducted a chi- square test among groups of students from various faculties, analyzing variables related to their interest in working in rural areas (Table 5).

**Table 5:** Descriptive statistics (frequencies and percentages) and chi-square test results for groups of students studying in Osijek (*N* = 57), Rijeka (*N* = 162), Split (*N* = 181), and Zagreb (*N* = 290) regarding their interest in working in rural areas.

**Variable**		**Osijek**	**Rijeka**	**Split**	**Zagreb**	***χ^2^***	***p***
Are you interested in a job outside the city center?	yes	36	94	97	159	2.041	0.564
		(63.2%)	(58.0%)	(53.6%)	(54.8%)		
	no	21	68	84	131		
		(36.8%)	(42.0%)	(46.4%)	(45.2%)		
Have you considered working in rural areas?	yes	35	82	96	173	4.795	0.187
		(61.4%)	(50.6%)	(53.0%)	(59.7%)		
	no	22	80	85	117		
		(38.6%)	(49.4%)	(47.0%)	(40.3%)		

There is no statistically significant difference in job interest outside city centers (χ^2^= 2.041; *P*= 0.564) or in considering rural medical practice (χ^2^= 4.795; *P* = 0.187) among medical students from the Universities of Osijek, Rijeka, Split, and Zagreb.

A statistically significant difference in the variables “job interest outside the (large) city center” and “considering working in rural areas,” depending on the place of origin from which the students come (Table 6).

**Table 6:** Descriptive statistics (frequencies and percentages) and chi-square test results for groups of students living in rural settlements (*N* = 136), smaller towns (*N* = 264), and larger towns (*N* = 290) regarding their interest in working in rural areas.

**Variable**		**Rural settlement (Village)**	**Smaller town**	**Larger town**	***χ^2^***	***p***
Are you interested in a job outside a large city center?	yes	98	160	128	33.058	0.000
		72.1%	60.6%	44.1%		
	no	38	104	162		
		27.9%	39.4%	55.9%		
Have you considered working in rural areas?	yes	93	139	154	10.647	0.005
		68.4%	52.7%	53.1%		
	no	43	125	136		
		31.6%	47.3%	46.9%		

Students from rural settlements were more often interested in jobs outside the city center compared to those from smaller towns [χ^2^(1) = 5.142, *p* = 0.023] and larger towns [χ^2^(1) = 28.978, *p* = 0.000]. Additionally, students from smaller towns more often interested in jobs outside the city center compared to those from larger towns [χ^2^(1) = 15.015, *p* = 0.000].

Also, students from rural settlements more often consider working in rural areas compared to students from smaller towns [χ^2^(1) = 9.118, *p* = 0.003] and larger towns [χ^2^(1) = 8.871, *p* = 0.003]. No statistically significant difference was found between students living in smaller towns and those in larger cities [χ^2^(1) = 0.11, *p* = 0.915].

## 4 Discussion

Compared to other studies, the results of this research indicate a stronger interest among students working in family medicine and outside urban centers, including in rural areas (42–47) and, given the gender structure of the sample and its comparison to the gender structure of the population of medical students in Croatia, this sample is representative (40). Other research findings show that students' choice of a medical profession is influenced by various factors, including personal and sociodemographic aspects, as well as different training programs and strategies (48, 49). As students mature from their first to final year of medical school, their experiences and acquired knowledge seem to significantly shape their professional choices. Medical school does not immediately equip students to practice independently. Instead, it provides a broad foundation of knowledge and skills that must be integrated and refined over time. Graduate students' interest in family and rural medicine is generally low (47, 50, 51) and there is a documented decline in interest from the first to the final year of study (48–55). The findings of the research however, are in contrast with existing literature, which is examined further in the discussion.

Responses to the first survey question, which explored students' willingness to pursue a career as family physicians), indicated that final-year students exhibited a greater inclination toward this career path compared to first-year students. The reasons for this difference are multifaceted and not fully understood, but likely factors include work conditions and the generalist-specialist divide. Hospital work differs significantly from family practice work in several respects. Hospital physicians typically operate in large, dynamic teams and environments, which allows for greater social interaction, peer consultation, and knowledge sharing, but also introduces more social stress and potential workplace harassment (56–60). Conversely, family physicians often work in smaller teams, typically comprising a doctor and a nurse or technician, which can offer a more controlled and less stressful work environment. Hospital physicians also face longer and more unpredictable working hours, including on-call duties and night shifts (61). Additionally, while hospital physicians tend to specialize in specific medical areas, family physicians require a broad knowledge base. Students who initially aim for a specialty might discover a preference for the comprehensive scope of general practice, while others may become more committed to their chosen specialty. A specific point in medical education in Croatia which could influence this outcome is that students get extensive practice in family medicine, with some medical schools requiring their students to do part of their practice in island settlements (24–27).

Tying into the differences between hospital and family practice work, the second question explored students' openness to working outside large medical centers. No significant difference was found between first-year and final-year students, with about half expressing openness to this idea. Hospitals, as the epicenters for complex patient care, allow physicians to engage with diagnostically and therapeutically challenging cases. Alternative career paths include general practice and private specialist clinics, with the latter offering more flexible working hours and often higher salaries, leading to a migration of physicians from hospitals to private clinics (62).

The third question, which showed the greatest difference between first-year and final-year students, examined their perceived readiness for rural practice upon graduation. While two-thirds of freshmen believed they would be adequately prepared, less than a third of final-year students agreed. This difference could partly be due to the recognition of the unique challenges rural environments present, such as limited support from the broader medical system and the greater difficulty of managing diagnostically uncertain cases which students would get the chance to see during their practice in island settlements. Moreover, the importance of hands-on experience becomes apparent during college; while medical school imparts essential skills, real- world experience is crucial for making sound clinical decisions (63).

As in many other countries, the curricula of medical faculties in Croatia do not have content related to rural medicine, nor special directions of education for working in rural areas (24–27). Educating students in a clinical environment has shown a higher level of satisfaction, knowledge, skills and attitudes among students, as well as a sense of readiness to assume the role of a doctor, stronger self-confidence and a more complex sense of professional identity (63). It could therefore be assumed that clinical exposure in rural environments would increase the students' attitude and willingness to work in rural areas (22, 23).

A near-universal opinion among students, regardless of their year, was that rural physicians face poor working conditions. This sentiment is not unique to Croatian students but is shared internationally (22, 23, 43, 45, 64). Several studies have examined if this truly is the case and have come out with complex results. The idea of working hours first comes up, as many physicians think that their rural colleagues have to work longer hours. Studies support this hypothesis, showing that rural physicians work on average 4 h longer than their city counterparts (65, 66). Patients in rural centers can be more medical challenging; they are on average older than those in the city, and have more disease complications due to postponing doctor visits because of the distance to the hospital (13). Whether rural family physicians provide a wider service profile to their patients is uncertain, with certain studies showing no difference with urban counterparts (65), while others show a wider service profile as well as more medical equipment use in rural practices (67). Despite these challenges, rural family physicians in America do not show a lower job satisfaction nor a higher burnout rate (68).

Financial compensation for rural physicians was supported by a majority of students from both groups, with more final-year students endorsing this view. It could be argued that due to longer working hours, more complex patients and lack of availability to the rest of the medical system, rural physicians should be paid more than their rural counterparts (13, 66, 67). Government programs rarely cite this as the reason they give financial incentives to rural physicians, but rather do it to attract doctors to underdeveloped areas. Poorer working conditions are not usually among the top reasons physicians, especially young doctors, avoid moving to rural centers; rather, they usually cite less opportunities for their family, be it work, school or entertainment (18).

An intriguing discovery was that the aspiration of students to work outside major city centers and in rural areas was consistent across the four universities studied. However, this desire varied based on the population size of the students' hometowns. Students from rural backgrounds were more inclined to work in rural areas compared to those from urban centers. This indicates that the inclination to work in rural areas is not influenced by the geographical location of the universities (which were distributed in urban centers across different regions of the country) but is instead linked to the students' backgrounds. These findings align with similar studies published in this field (17, 18).

Despite believing they are not prepared, not well compensated and will work in inadequate conditions, final year students still show more willingness to work in rural areas. A potential reason for the interest expressed by students in Croatia in working in rural areas, as previously mentioned, may be the point-based system for work experience used in applications for specialization. Further studies in this area could be valuable to establish what factors draw students to work in rural areas, especially in light of the three main findings of this research: student's rural background, financial incentives and improved working conditions. Other papers in this area suggest that financial incentives alone may not suffice to attract physicians to rural areas; improving rural infrastructure and creating more opportunities for employment and community development, together with enabling contact with rural medicine during medical school, might prove more effective in addressing the shortage (14–17).

## 5 Conclusion

Croatian medical students express interest in working in rural areas; however, their education does not adequately prepare them for this practice. Final-year students are more willing to become family physicians and work in rural areas despite believing that medical school does not adequately prepare a student for rural work. Students originally from rural settlements are especially interested in working in rural areas in the future. Additionally, they believe that current working conditions for rural doctors are inadequate and that these professionals should receive further incentives.

### 5.1 Implication

The results of this research provide a compelling argument for enhancing medical school curricula by incorporating rural medicine content and strengthening the practical components of rural medical education in Croatia. As a pilot study, it provides preliminary insights into students' attitudes toward family practice and rural medicine. Future research could take a longitudinal approach to track how these attitudes evolve over time. Additionally, studies exploring students' perceptions of their rural education, along with intervention studies evaluating changes to the curriculum, would be valuable.

### 5.2 Strengths and limitations of the study

The findings from this research have practical implications for the educational program, particularly in terms of increasing the focus on rural medicine.

However, it is important to acknowledge the limitations of this study. The research was conducted online, which raises the possibility that the participants who chose to take part may differ from the general population of medical students. This is a limitation because online participants may have distinct characteristics, such as greater access to technology or different levels of motivation, potentially introducing selection bias and limiting the generalizability of the findings to the broader population of medical students. Another limitations is the study's cross-sectional design which limits the ability to make causal relations between the changes students go through (educational, personal or maturational) and their opinions on rural and family medicine.

## Data Availability

The raw data supporting the conclusions of this article will be made available by the authors, without undue reservation.
